# Combined *Rothia dentocariosa* and *Streptococcus viridans* Corneal Ulcer in an Immunocompromised Patient

**DOI:** 10.1155/2021/9014667

**Published:** 2021-11-16

**Authors:** Jamie Dietze, Thomas Mauger

**Affiliations:** WVU Medicine Department of Ophthalmology, USA

## Abstract

Keratitis is a very common condition seen by ophthalmologists. However, many factors can complicate the treatment of this depending on the causative organism and other patient comorbid conditions. The objective of this clinical case report is to highlight the treatment of keratitis caused by *Rothia dentocariosa*. It also looks at the unique considerations in keratitis presentations for patients immunocompromised by chemotherapy agents. Our patient is a 58 yo female undergoing chemotherapy with folinic acid, fluorouracil, irinotecan, and panitumumab who presented with several days of a red, painful right eye with mucous discharge. Cultures were positive for *Rothia dentocariosa* and *Streptococcus viridans*. The patient ultimately underwent a conjunctival flap procedure as medical therapy with proper oral and topical antibiotics failed to resolve keratitis. This case is unique as previously, only a couple of cases of keratitis caused by *Rothia dentocariosa* have been reported and none of those patients were immunocompromised nor failed antibiotic therapy.

## 1. Introduction

*Rothia dentocariosa* is a gram-positive rod that was originally isolated from dental plaques and caries. It is commonly found in the oral cavity and pharynx as part of the normal microflora [1]. It has also been a source for transient bacteremia, endocarditis, and some meningitis, peritonitis, bone/joint infections, pneumonia, skin/soft tissue, endophthalmitis, and prosthetic device infections. Invasive disease of *Rothia* species occurs predominantly in immunocompromised hosts, and patients typically have periodontal disease or multiple carious teeth [2]. It is usually susceptible to penicillin, cephalosporin, erythromycin, and tetracycline, but there have been a few strains found that have developed B-lactamase [2]. Reported ocular infections of *Rothia* species typically are endophthalmitis following intravitreal injection or systemic bacteremia seeding [3]. We describe a case of keratitis due to *R. dentocariosa* in an immunocompromised host.

## 2. Case

**Figure 1:**
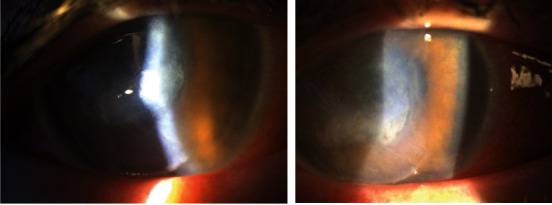
Slit lamp photos showing keratitis of patient's right eye three days after initial presentation. Central opacification with peripheral corneal clearing and diffuse conjunctival injection.

**Figure 2:**
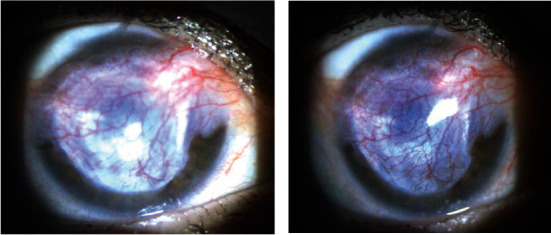
Slit lamp photos showing patient's right eye six weeks postoperatively from conjunctival flap placement. The flap appears to be in proper placement over previous corneal defect with good vascularization.

A 58-year-old white female presented with a red, painful right eye. She had some photosensitivity and increasingly blurry vision. Her history was significant for daily wear soft contact lens use and stage IV colon cancer treated with partial bowel resection and was currently undergoing chemotherapy FOLFIRI (folinic acid, fluorouracil, and irinotecan) and panitumumab every other week. The exam was significant for hand motion visual acuity and moderate diffuse conjunctival injection with moderate mucous discharge (Figure 1). The cornea was diffusely opaque in the central 8 mm and an overlying epithelial defect. The far periphery had less dense anterior stromal corneal haze. The remainder of the exam was limited due to poor view into the anterior chamber. Ultrasound B scan showed no evidence of vitritis or endophthalmitis. Cultures for fungus, bacteria, and atypical microorganisms were taken, and the patient was started on fortified vancomycin (50 mg/ml) and fortified tobramycin (15 mg/ml) every hour, moxifloxacin every two hours, cyclopentolate three times per day, ciprofloxacin 500 mg by mouth twice daily, and vitamin C 500 mg by mouth twice daily. Bacteria cultures grew *Rothia dentocariosa* with sensitivities showing resistance to erythromycin and intermediate sensitivity to penicillin. *Strep viridans* also grew in the bacterial culture (Table 1). The treatment regimen was switched to moxifloxacin four times per day, fortified tobramycin four times per day, vancomycin every hour, cyclopentolate three times per day, vitamin C 500 mg by mouth twice daily, and doxycycline 100 mg by mouth twice daily. The patient continued to have an 8 mm epithelial corneal defect with irregular borders and central granular infiltrates but peripheral corneal clearing. The regimen was changed to fortified vancomycin every two hours, moxifloxacin every two hours, cefuroxime 50 mg/ml every hour while awake, and acyclovir 400 mg by mouth five times daily. The patient did not show further improvement, so cefixime 400 mg by mouth daily was added and acyclovir and doxycycline were discontinued. After six days on this regimen and still without significant improvement, the patient underwent a Gunderson conjunctival flap. A flap was taken from the superior bulbar conjunctiva and rotated over the cornea secured with 8-0 Vicryl sutures. The patient showed gradual improvement in symptoms, and the ocular inflammation was resolved. Six weeks postoperatively, the infection appeared to be resolved and the flap appeared to be well healed (Figure 2). The patient had been off antibiotic drops for four weeks, only using erythromycin ointment, and had total resolution of her ocular pain as well as no signs of recurrent infection. There is plan for keratoplasty in the future.

## 3. Discussion

Georg and Brown first created the genus “Rothia” in 1967, which resembles both Nocardia and Actinomyces but differs in the physiology and cell wall constituents [4]. *Rothia dentocariosa* is a rod-shaped, nonsporogenic, nonmotile, catalase-positive, gram-positive bacterium that grows faster under aerobic rather than anaerobic conditions but can grow in either environment. Its colonies are either smooth and convex or rough with a “spoke-wheel” surface with scalloped edges [5]. *R. dentocariosa* is considered largely benign but can rarely cause infection, most commonly endocarditis in people with heart valve disorders. Typically, patients who get infections are predisposed by having poor dental hygiene or have had extensive dental procedures [1].

*Rothia* species tend to be widely susceptible to the penicillin class and other antibiotics covering gram-positive bacteria. However, there have been some recent reports of species with B-lactamase activity, therefore causing some penicillin resistance [6]. Despite this, the current accepted treatment for *Rothia* infections such as endocarditis is still treatment with penicillin [7].

Our patient presented with keratitis without any evidence of endophthalmitis. There have only been two previous cases reported of *R. dentocariosa* causing corneal ulceration. First was an 11-year-old boy with history of keratoplasty and was found to be licking his finger prior to eye rubbing [8]. The second was a 49-year-old South Asian female with history of recent dental procedure and a canine that frequently licked her face [9]. Both cases were treated with a cephalosporin and resolved. Our case, however, is the first one reported in an immunocompromised host that did not have a significant dental history.

*Streptococcus viridans* is an alpha group streptococcus that is a known pathogen for causing corneal ulceration and infectious crystalline keratopathy, especially in postkeratoplasty patients. It is thought to be of low-grade pathogenicity as it infects locally immunosuppressed hosts or those with surface damage due to contact lens wear. Infections are typically indolent, causing an anterior stromal inflammatory reaction that is slow to progress with or without a mild anterior chamber reaction. When ulceration occurs, it is typically a well-circumscribed, gray white, “dry” appearing, and lying beneath a well-demarcated epithelial defect [10]. When seen as a crystalline keratopathy, it appears as discrete, white, crystalline/fernlike stromal opacities under an intact epithelium with little corneal inflammation. Typically, infections with *S. viridans* respond rapidly to proper antibiotic therapy [10]. *S. viridans* has continued to have good susceptibility to vancomycin in recent literature and is still considered a proper antimicrobial therapy for ocular infections [11, 12]. There have not been any reports of ocular coinfections with *Rothia* species, but *S. viridans* has been known to have coinfection with fungus, such as Candida and Acanthamoeba [13, 14]. We believe our patient had a true coinfection with *R. dentocariosa* and *S. viridans* rather than a superinfection based on her symptom timeline and the initial culture growing both species.

Our patient was immunocompromised due to ongoing FOLFIRI and panitumumab chemotherapy treatment. Panitumumab is one of the Epidermal Growth Factor Receptor (EGFR) inhibitor chemotherapies. EGFR inhibitors have been shown to cause poor healing of the corneal epithelial layer, corneal thinning, and melt. Some other chemotherapy agents can cause corneal deposits, verticillata, and tear film changes leading to both corneal and conjunctival epithelial damage [15]. While we know EGFR inhibitors can affect the corneal tissues, the actual mechanism is unclear as to which endogenous EGF-like ligand promotes corneal wound healing and therefore is disrupted by EGFR inhibitors. What is known is that having high level of basal EGF in tear fluid promotes EGFR-mediated corneal epithelial homeostasis and is the only ligand that is present in concentrations that would predict a significant level of receptor occupancy and therefore is the most likely mechanism that EGFR inhibitors influence the cornea [16].

Another aspect of interest about our patient was that the *R. dentocariosa* species was found to be erythromycin resistant and only intermediately sensitive to penicillin. She was treated with cefixime PO, ceftazidime drops QID, and vancomycin drops QID but only showed modest ocular improvement. She ultimately required a conjunctival flap to promote corneal healing, which was not needed in either of the previous two cases.

Conjunctival flaps were first started in the 1800s and used for corneal ulcerations and thinning disorders. Their purpose is to restore the integrity of the compromised corneal surface and provide metabolic and mechanical support for corneal healing [17, 18]. They can be used in cases where medical treatment has been exhausted to resolve infection such as our patient but are usually used for cases with marginal fungal ulcers but have also been shown to benefit in other infectious keratitis such as Acanthamoeba [19]. Flaps help improve comfort and ocular inflammation and promote healing [20]. Vascularized conjunctival tissues give both a rich blood supply and lymphatic vessels providing better nutrition to the corneal tissues, growth factors, an increased resistance to infection, and anticollagenolytic substances [17]. As long as the graft continues to stay healthy, these can be left in place. Once infection resolution is achieved, keratoplasty may be performed. Using a conjunctival flap as an intermediate step can lower the risk of keratoplasty graft rejection as compared to cases that do not use a conjunctival flap temporizing measure [20]. That is why a conjunctival flap intermediate step was chosen for our immunocompromised patient, so that she could continue chemotherapy treatment but reduce the risk of infection recurrence and graft failure of a keratoplasty procedure.

## 4. Conclusion

*Rothia dentocariosa* is a known part of the normal microflora of the oropharynx. It typically does not cause invasive disease, but when it does, it tends to cause endocarditis or pneumonia. Ocular infections with this organism are typically thought to be limited to post procedure endophthalmitis. Corneal ulceration is a rare presentation of *R. dentocariosa* infection and should be considered on the differential diagnosis in patients with recent extensive dental procedures and poor dental hygiene or those who are immunocompromised. While *R. dentocariosa* is still considered widely susceptible to penicillin, some resistance is starting to emerge. Treatment with a cephalosporin is the current recommendation for systemic infection when resistance is detected and therefore should be considered the treatment for ocular infection. In cases where ulceration or keratitis persists despite cephalosporin treatment, a conjunctival flap may be of benefit to resolve the infection prior to a keratoplasty procedure.

## Figures and Tables

**Table 1:** Antibiotic sensitivity table from cornea bacterial culture performed.

Rothia dentocariosa		
Ceftriaxone	1 mcg/ml	Sensitive
Erythromycin	4 cg/ml	Resistant
Penicillin		Intermediate
Vancomycin	0.5 mcg/ml	Sensitive
